# Apical Acute Myocardial Infarction Due to Occluded Posterior Descending Branch of Right Coronary Artery Concomitant With Short Left Anterior Descending Artery: Multi-imaging Modality Assessment

**DOI:** 10.7759/cureus.64485

**Published:** 2024-07-13

**Authors:** Satoshi Kurisu, Hitoshi Fujiwara

**Affiliations:** 1 Department of Cardiology, National Hospital Organization Hiroshimanishi Medical Center, Otake, JPN

**Keywords:** cardiac imaging, electrocardiogram (ecg/ekg), coronary tree, resting echocardiography, myocardial edema

## Abstract

Regional wall motion abnormality in the left ventricular (LV) apex detected on transthoracic echocardiography is commonly interpreted as the presence of a distal left anterior descending (LAD) artery lesion in clinical practice. Herein, we reported a rare case of apical acute myocardial infarction (AMI) caused by an occluded posterior descending branch of the right coronary artery (RCA), in which the correspondence between coronary arterial anatomy and supplied LV apex was evaluated by multi-imaging modalities.

Despite the presence of regional wall motion abnormality in the LV apex, left coronary angiography showed no significant coronary artery diseases. It was of note that LAD terminated before the LV apex. Right coronary angiography showed total occlusion of the posterior descending branch. Cardiac computed tomography (CT) clearly demonstrated that the spontaneously recanalized posterior descending branch extended toward the LV apex. Cardiac magnetic resonance imaging (MRI) clearly revealed regional wall motion abnormality corresponding to myocardial edema in the LV apex. Cardiac CT and MRI were powerful tools in clarifying the correspondence between coronary arterial anatomy and supplied LV apex. Clinicians should be aware that localized apical AMI can occur under the condition of occluded posterior descending branch of RCA concomitant with short LAD.

## Introduction

A diagnosis of acute myocardial infarction (AMI) is made from a combination of clinical history, electrocardiographic (ECG) changes, and cardiac troponin release [1]. However, some patients may have unstable coronary artery disease without significant ECG changes or troponin release [2]. Given that loss of function is one of the characteristics of the myocardial area at risk, transthoracic echocardiography (TTE) is commonly performed as the first-line imaging modality to evaluate cardiac structure as well as function in patients with suspected AMI. The left anterior descending artery (LAD) is generally considered to be the primary vessel supplying the left ventricular (LV) apex [3]. For this reason, regional wall motion abnormality in the LV apex detected on TTE is commonly interpreted as the presence of distal LAD lesions in clinical practice.

Recent advances in cardiac imaging, such as cardiac computed tomography (CT) and magnetic resonance imaging (MRI), have allowed for comprehensive assessment of AMI noninvasively.

Herein, we reported a rare case of apical AMI an caused by occluded posterior descending branch of the right coronary artery (RCA), in which the correspondence between coronary arterial anatomy and supplied LV apex was evaluated by multi-imaging modalities.

## Case presentation

An 82-year-old woman with hypertension, who had been treated with telmisartan (40 mg) and cilnidipine (20 mg), presented to the emergency department with a chest oppressive sensation persisting for one hour at night.

On physical examination, her pulse rate was 82 bpm, blood pressure was 184/70 mmHg, and oxygen saturation was 96%. There were no audible murmurs. Creatine kinase (CK)-MB and troponin-I values were slightly increased. Renal function was normal (Table 1).

**Table 1:** Laboratory data CK, creatine kinase

Variable	Initial presentation	9 hours later	3 days later	Reference range
White blood cell counts	7.7 × 10^3^ cells/mm^3^			3.3 - 8.6 × 10^3^ cells/mm^3^
Red blood cell counts	4.30 × 10^6^ cells/mm^3^			3.86 - 4.92 × 10^6^ cells/mm^3^
Hemoglobin	12.2 g/dL			11.6 - 14.8 g/dL
Platelet counts	206 × 10^3^ cells/mm^3^			158 - 348 × 10^3^ cells/mm^3^
Aspartate aminotransferase	20 U/L	31 U/L	49 U/L	13 - 30 U/L
Alanine aminotransferase	10 U/L	10 U/L	14 U/L	7 - 23 U/L
Lactate dehydrogenase	210 U/L	202 U/L	279 U/L	124 - 222 U/L
CK	81 U/L	176 U/L	256 U/L	41 - 153 U/L
CK-MB	9.2 U/L	13.7 U/L		0 - 5.0 U/L
Troponin-I	29 pg/mL			0 - 26.2 pg/mL
Blood urea nitrogen	17.3 mg/dL			8 - 20 mg/dL
Creatinine	0.77 mg/dL			0.46 - 0.79 mg/dL
Low-density lipoprotein cholesterol		123 mg/dL		65 - 163 mg/dL
High-density lipoprotein cholesterol		65.1 mg/dL		48 - 103 mg/dL
C-reactive protein	0.04 mg/dL			0 - 0.14 mg/dL
N-terminal pro-brain natriuretic peptide	171 pg/mL			0 - 126 pg/mL

**Figure 1:**
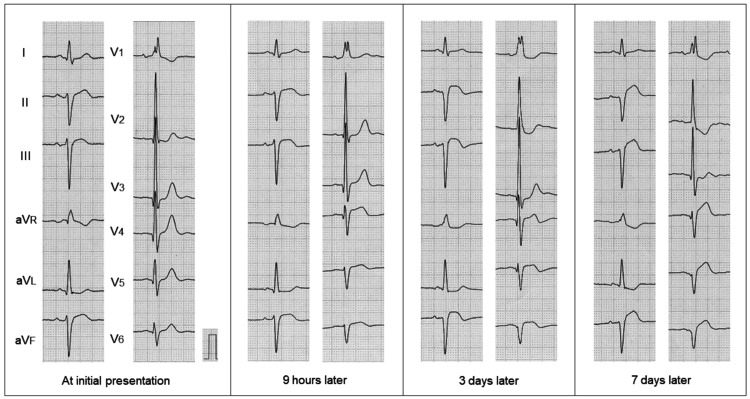
Serial ECG ECG revealed a right bundle branch block with left axis deviation. ST segment elevations in inferior leads were marginal at the initial presentation. Nine hours after admission, follow-up ECG showed significant ST segment elevations in inferior leads and decreased R wave amplitudes in V_4-6_ leads. ECG, electrocardiogram

An electrocardiogram (ECG) revealed a right bundle branch block with left axis deviation (Figure 1). ST segment elevations in inferior leads were marginal at that time. A chest radiograph showed no pulmonary congestion. The patient was admitted for further cardiac examinations.

**Figure 2:**
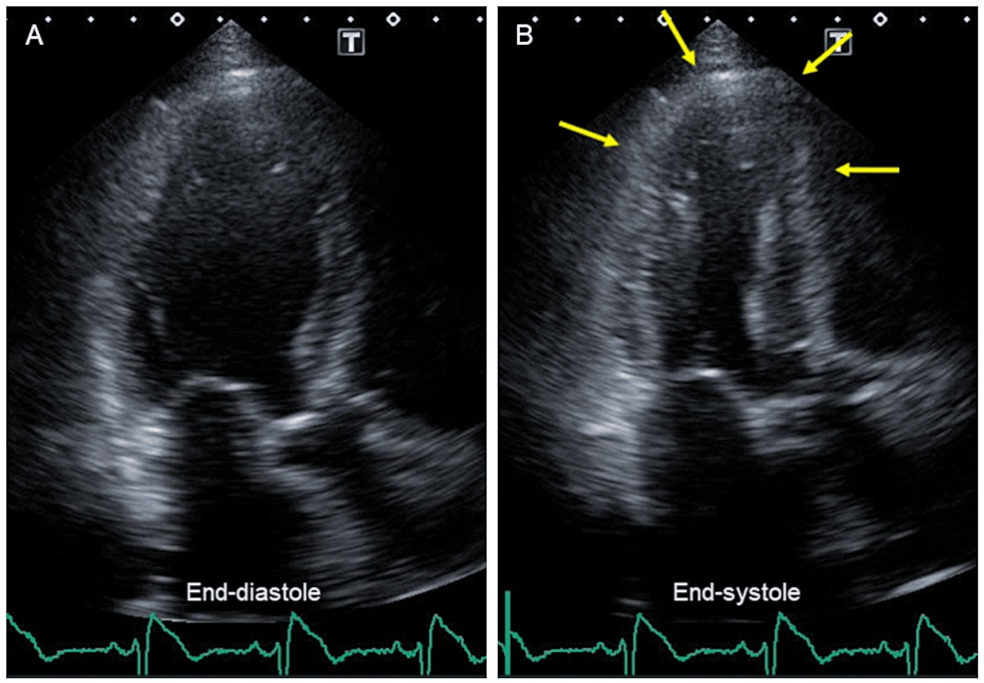
Echocardiogram A TTE showed regional wall motion abnormality in the LV apex with an ejection fraction of 63% (arrows). LV, left ventricular; TTE, transthoracic echocardiogram

Nine hours after admission, laboratory data revealed further increased CK and CK-MB values although the patient’s symptoms disappeared (Table 1). Follow-up ECG showed significant ST segment elevations in inferior leads and decreased R wave amplitudes in V_4-6_ leads (Figure 1). A TTE showed regional wall motion abnormality in the LV apex with an ejection fraction of 63% (Figure 2; arrows). No significant valvular heart diseases were found.

**Figure 3:**
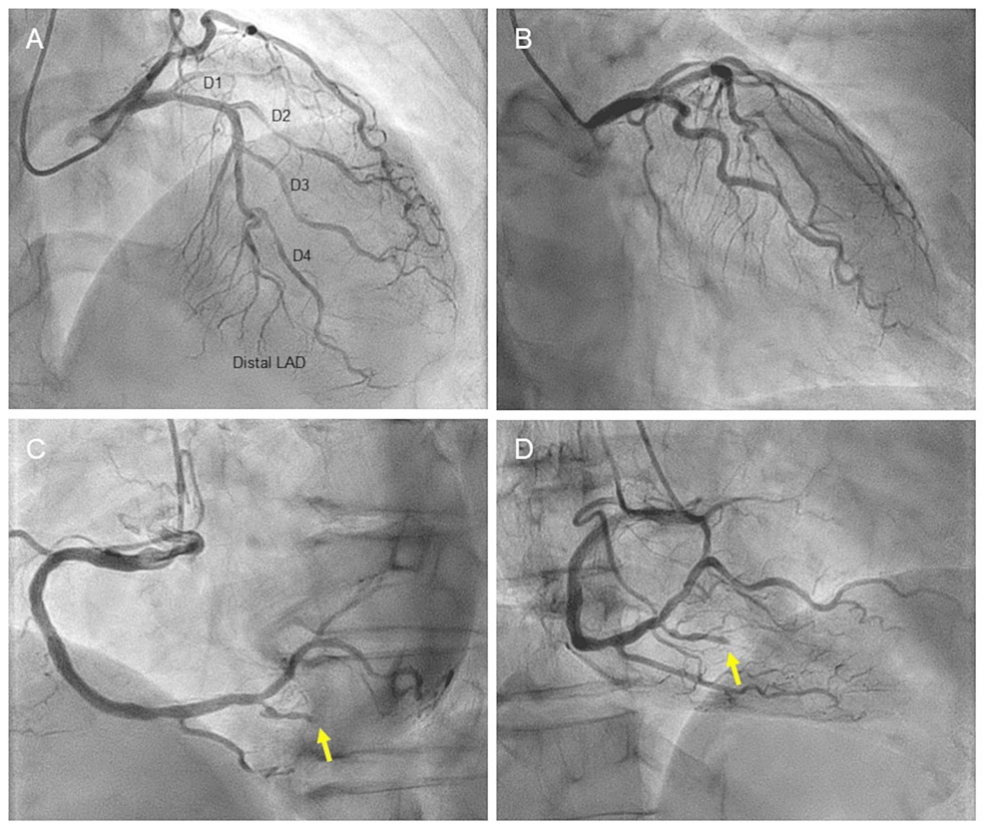
Left and right coronary angiograms Despite the presence of regional wall motion abnormality in the LV apex, left coronary angiography showed no significant coronary artery diseases (A and B). It was of note that the LAD artery terminated before the LV apex. Right coronary angiography showed total occlusion of the posterior descending branch (C and D, arrows). LAD, left anterior descending; LV, left ventricular

Because of the high possibility of AMI, coronary angiography was performed through the right radial artery. Despite the presence of regional wall motion abnormality in the LV apex, left coronary angiography showed no significant coronary artery diseases (Figures 3A, 3B). It was of note that LAD terminated before the LV apex. Right coronary angiography showed total occlusion of the posterior descending branch (Figures 3C, 3D; arrows). No collateral circulation was found.

The patient was diagnosed with localized apical AMI associated with a distal RCA lesion. Given that the culprit lesion was located at the distal end of RCA, the coronary intervention was not indicated. The patient was treated conservatively with carvedilol (2.5 mg/day), clopidogrel (75 mg/day), and rosuvastatin (2.5 mg/day).

**Figure 4:**
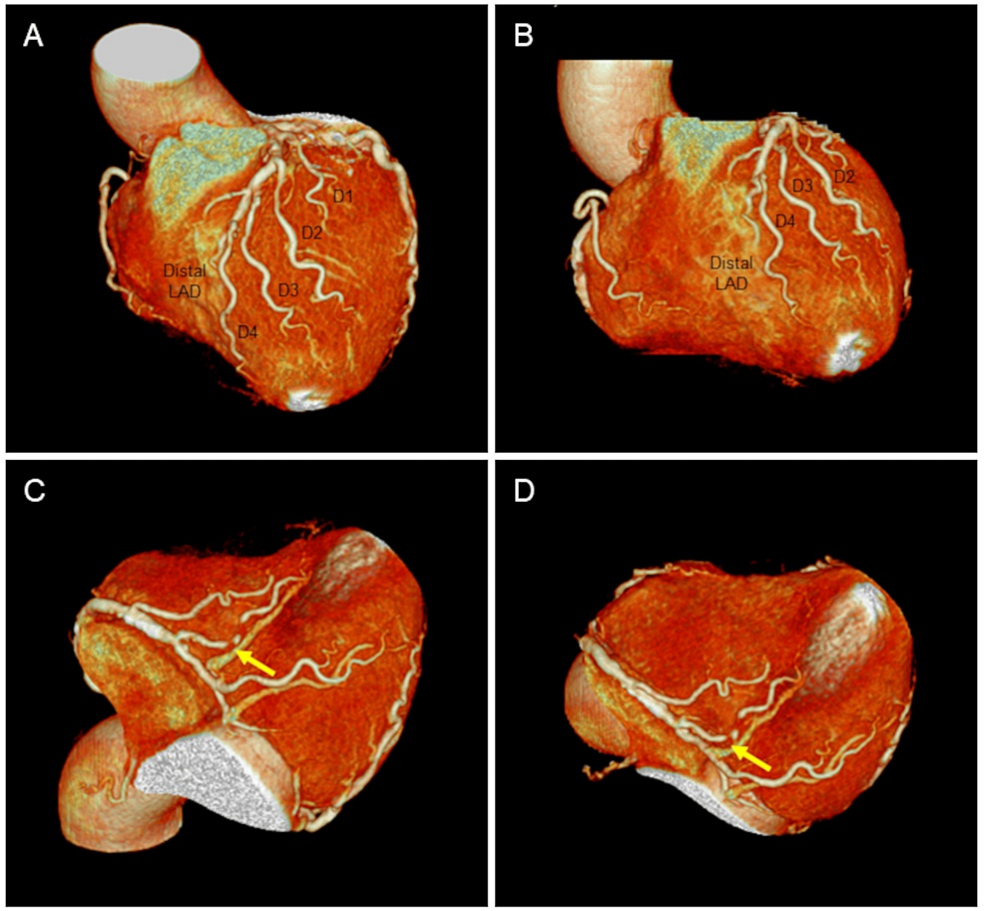
Cardiac CT LAD artery after takeoff of the fourth major diagonal branch (D4) terminated before the LV apex (A and B). As for the right coronary artery, the posterior descending branch was spontaneously recanalized, and its distal end extended toward the LV apex (C and D, arrows). LAD, left anterior descending; LV, left ventricular; CT, computed tomography; RCA, right coronary artery

On hospital day 3, cardiac CT was performed to evaluate the correspondence between coronary arterial anatomy and supplied LV apex. LAD after takeoff of the fourth major diagonal branch terminated before the LV apex (Figures 4A, 4B). As for RCA, the posterior descending branch was spontaneously recanalized, and its distal end extended toward the LV apex (Figures 4C, 4D; arrows).

**Figure 5:**
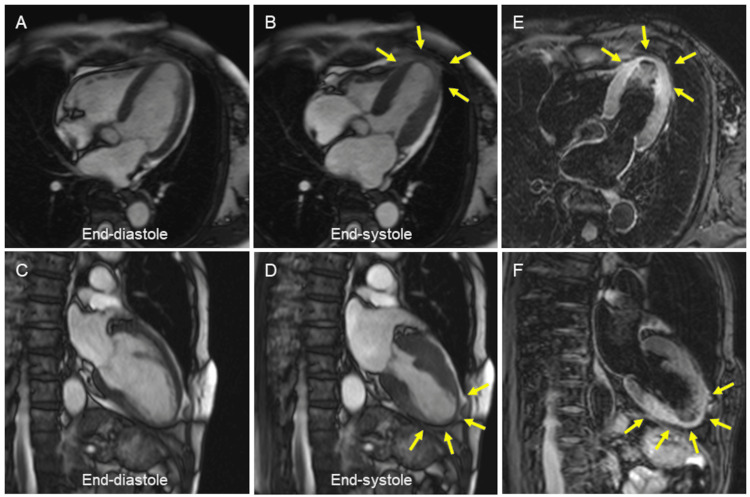
Cardiac MRI Cine MRI revealed regional wall motion abnormality in the LV apex (A to D, arrows), where T2-weighted MRI detected myocardial edema, indicative of the myocardial area at risk (E and F, arrows). LV, left ventricular; MRI, cardiac magnetic resonance imaging

On hospital day 5, a cardiac MRI was performed to identify the myocardial area at risk. Cine MRI revealed regional wall motion abnormality in the LV apex (Figures 5A-5D; arrows), where T2-weighted MRI detected myocardial edema, indicative of the myocardial area at risk (Figures 5E, 5F). Myocardial edema extended further into the LV mid-inferior wall with no wall motion abnormality. There was no significant gadolinium enhancement in the LV apex.

No adverse cardiac events occurred during hospitalization and the patient was discharged seven days later. The patient remained in good condition without recurrent ischemic attacks.

## Discussion

In this report, we presented a case of apical AMI caused by an occluded posterior descending branch of RCA concomitant with short LAD. The combination of cardiac CT and MRI was useful in clarifying the correspondence between coronary arterial anatomy and supplied LV apex.

Regional wall motion abnormality in the LV apex is generally assumed to result from distal LAD lesions because this vessel is the usual source of blood supply to the LV apex. Several angiographic studies showed anatomical variations in the blood supply to the LV apex [4,5]. Perlmutt et al. included 431 patients undergoing coronary angiography, revealing that LV apex received blood supply by the LAD in 78%, and dual blood supply from both the LAD and posterior descending branch of RCA in 12% of the patients. In 10%, the LAD terminated before the LV apex, which received blood supply from the posterior descending branch [4]. However, such angiographic studies have limitations in identifying the vessels supplying the LV apex. Ortiz-Perez et al. included 93 patients with AMI who underwent both coronary angiography and cardiac MRI and investigated the correspondence between the coronary arterial anatomy and supplied myocardium [6]. They demonstrated that the positive predictive value of the presence of hyper-enhancement in the LV apex was 91% for LAD, whereas it was only 4% for RCA. Their results suggest that localized apical AMI associated with RCA is rare.

In our case, we first obtained coronary angiograms. However, it was difficult to confirm the correspondence between coronary arterial anatomy and supplied LV apex in part because the distal segment beyond the occlusion of the posterior descending branch was not seen. This was the reason why further cardiac imaging studies were performed. Cardiac CT clearly demonstrated that the spontaneously recanalized posterior descending branch extended toward the LV apex [7,8]. Cardiac MRI is another useful imaging modality for the assessment of LV function and myocardial perfusion during a single examination [9]. The bright signal on the T2-weighted MRI suggests myocardial edema, thereby exhibiting the myocardial area at risk in AMI [10]. In our case, MRI clearly revealed regional wall motion abnormality corresponding to myocardial edema in the LV apex.

There have been few reports on ECG in localized apical AMI in part due to its low incidence in clinical practice. Fiorina et al. evaluated ECG criteria of apical AMI as proposed in the literature in patients with apical perfusion defect on scintigraphy and found their low diagnostic sensitivities [11]. Savage et al. evaluated the correlation of postmortem anatomic findings with ECG changes in patients with AMI [12]. They showed that eight of the 12 anterior AMIs exhibited circumferential apical involvement, being associated with Q waves or markedly diminished R waves in the left precordial leads. Consistent with their results, our patient exhibited diminished R waves in V_4-6_ leads during hospitalization.

Except for distal LAD lesions, takotsubo syndrome, hypertrophic cardiomyopathy with apical aneurysm, and distal RCA lesion are possible conditions of regional wall motion abnormality in the LV apex. Especially in elderly patients, invasive coronary angiography may not be feasible for a variety of reasons. The present case suggested that the combination of cardiac CT and MRI is useful for differential diagnosis as an alternative to coronary angiography.

## Conclusions

In conclusion, we encountered a case of localized apical AMI associated with a distal RCA lesion. Cardiac CT and MRI were powerful tools in clarifying the correspondence between coronary arterial anatomy and supplied LV apex. Clinicians should be aware that localized apical AMI can occur under the condition of occluded posterior descending branch of RCA concomitant with short LAD. This should contribute to the clinical management of patients presenting with regional wall motion abnormality in the LV apex.
